# Burden of unmet health-related social needs in an academic adult primary care practice in San Francisco California

**DOI:** 10.1186/s12875-023-02125-2

**Published:** 2023-08-25

**Authors:** Jane Jih, Antony Nguyen, Irena Cenzer, Jennifer Morrish

**Affiliations:** 1 Division of General Internal Medicine, Department of Medicine, University of California San Francisco, 490 Illinois St, San Francisco, CA 94158 USA; 2 Asian American Research Center on Health, 490 Illinois St, San Francisco, CA 94158 USA; 3 Multiethnic Health Equity Research Center, University of California San Francisco, 490 Illinois St, San Francisco, CA 94158 USA; 4 Division of Geriatrics, Department of Medicine, University of California San Francisco, 4150 Clement St, San Francisco, CA 94121 USA; 5 University of California San Francisco Health, 1545 Divisadero St, San Francisco, CA 94143 USA

**Keywords:** Health-related social needs, Primary care, Social care, Health equity, Health disparities

## Abstract

**Objectives:**

Unmet health-related social needs can influence health outcomes and increase healthcare utilization. There is growing interest in integrating social needs care into healthcare delivery. We conducted an assessment of health-related social needs in an academic adult primary care practice in San Francisco, California.

**Methods:**

We recruited a random convenience sample of adult English-, Chinese- or Spanish-speaking patients from clinic waiting rooms at the study sites to complete a self-administered, anonymous survey. We used the Accountable Health Communities Health-Related Social Needs Screening Tool for these domains: housing instability, food insecurity, transportation problems, utility help needs, interpersonal safety, financial strain, and family/community support. We conducted univariate and multivariate analyses adjusting for age, sex and survey language.

**Results:**

679 patients completed the survey. Respondents were 57% female and mean age of 58 ± 18 years old. 54% of patients had at least one unmet health-related social need. The most prevalent health-related social needs were financial strain (35%), at least one issue with housing conditions (27%), and food insecurity (23%). Respondents completing the survey in Spanish had significantly higher odds of reporting food insecurity (AOR 3.97, 95%CI 1.86, 8.46), transportation problems (AOR 3.13, 95%CI 1.32, 7.43), and need for support with activities of daily living (AOR 4.58, 95%CI 2.04, 10.25) than respondents completing the survey in English.

**Conclusions:**

The burden of unmet health-related social needs was considerable in this adult primary care practice. These findings can support a case for integrating health-related social need screening and social care in the delivery of primary care in the United States to advance health equity.

## Introduction

Unmet health-related social needs (HRSNs), such as housing instability, food insecurity, and transportation challenges, can influence both the delivery and utilization of healthcare and health outcomes [1–7]. Identifying and addressing the unmet social needs of patients in clinical settings could not only lower healthcare utilization and cost but could allow for delivery of healthcare that is more person-centered and result in more equitable health outcomes [7–9]. Integrating social needs care including HRSN screening into healthcare delivery in the United States (U.S.) is an emerging aspect of clinical practice but not yet part of routine care [6, 7, 10, 11].

Primary care clinics are one clinical setting in which HRSN screening could occur. There is limited literature on the screening of multiple unmet HRSNs within adult primary care settings caring for linguistically diverse patients in the U.S [12]. As about 8% of people in the U.S. are limited English proficient (defined as self-report of speaking English “not well” or “not at all”) [13] and to promote health equity and to not augment known health inequalities for non-English speakers, [14–16] inclusion of patients whose primary language is not English is important. To redesign primary care delivery and infrastructure to support HRSN screening and social needs care, understanding the burden of HRSN in primary care settings is an essential step. With this in focus, we conducted a one-time HRSNs assessment to examine the burden of unmet HRSNs in a large, diverse, urban, academic adult primary care practice that was not otherwise systematically screening and collecting social need data.

## Methods

We conducted this cross-sectional observational study at a three-site urban academic adult primary care practice in San Francisco, California that serves a multiethnic and linguistically diverse patient population of over 25,000 patients including 21% Asian American, 9% Black/African American, and 8% Latinx; the most prevalent non-English languages were Chinese (Cantonese (2.5%) and Mandarin (1.1%)) followed by Spanish (1.3%). The study period was February to October 2019. At the time in which the study was conducted, HRSNs were not systematically assessed at the study sites. The University of California San Francisco institutional review board reviewed the study procedures and provided this project with exempt certification. We sought feedback on the study procedures from practice leadership and the practice’s Patient Advisory Council.

### Study procedures

Bilingual research assistants (English/Cantonese, English/Mandarin and English/Spanish) independently approached a convenience sample of patients awaiting appointments in clinic waiting rooms at each of the three-sites to complete a self-administered, anonymous survey in English, Chinese or Spanish. As the primary focus of this project was to do a needs assessment of unmet social needs, we sought to recruit at least 250 patients from each of the larger two sites and at least 100 patients from the smaller third site with a secondary aim to oversample Chinese- and Spanish-speakers for multivariable analyses. Patients were eligible to complete the survey if (1) they spoke or read English, Chinese (Cantonese and Mandarin) or Spanish; and (2) had a same day appointment. Patients were invited to complete the survey on paper or through an online Research Electronic Data Capture (REDCap) survey on a study iPad or their own smartphone device prior to their clinic visit. Patients’ caregivers were permitted to assist patients in completing the survey as needed. Research assistants were trained to approach all potentially eligible patients in the waiting room during randomly selected clinic half-days recruitment sessions. During these half-day recruitment sessions, we also displayed in-language signs in the waiting rooms promoting the survey to invite patients to approach research assistants if interested in participating.

Each participant received a $5 gift card incentive for completing the survey and was offered a printed guide in English, Chinese or Spanish with local resources for all the HRSN domains included in the survey. The HRSN resource guide was compiled and refined by an interdisciplinary team including the licensed clinical social worker at the study sites. Patients were encouraged by research assistants to utilize the printed resources guide to fulfill their unmet needs. The printed resource guide also encouraged the patient to discuss any concerns raised by the survey with their clinician during the visit. Patient responses were inputted into REDCap.

### Assessment of health-related social needs

**Fig. 1:**
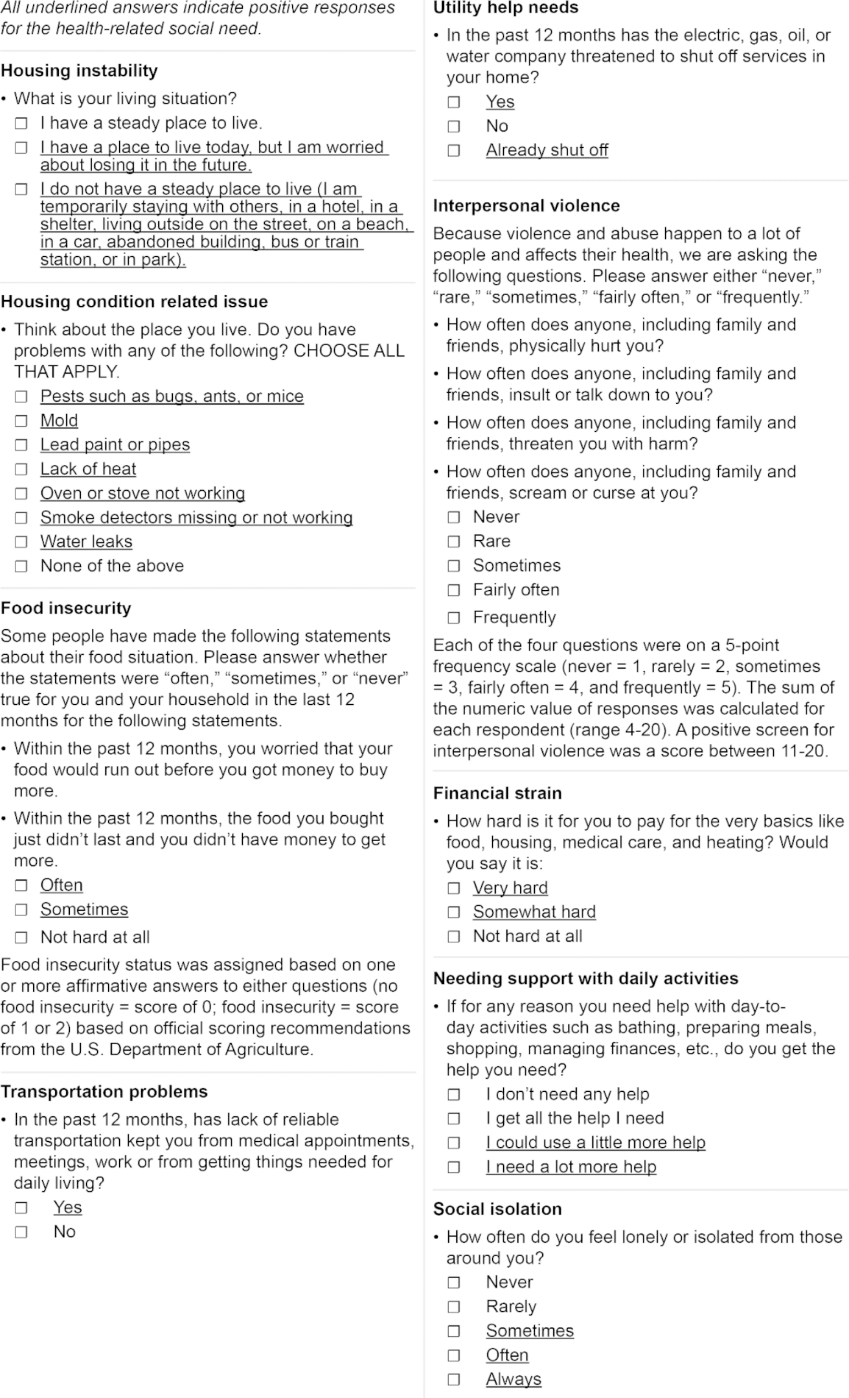
Unmet Health-Related Social Needs Assessment Survey. The survey was adapted from the Accountable Health Communities Health-Related Social Needs Screening Tool by the Center for Medicare and Medicaid Services and translated into Chinese and Spanish for use

The survey was composed of 13 questions from the Accountable Health Communities Health-Related Social Needs Screening Tool [17] for these domains presented in Fig. 1: housing-related problems (two questions), food insecurity (two questions), transportation problems (one question), utility help needs (one question), interpersonal safety (four questions), financial strain (one question), and family and community support (two questions). We also asked age, sex, and zip code of residence; no additional sociodemographic or health characteristics were collected. Aside from the food insecurity survey questions in Chinese and Spanish provided by the U.S. Department of Agriculture that have undergone validation and refinement, [18, 19] all remaining survey questions were translated into traditional Chinese and Spanish language by a bicultural, bilingual team member and then reviewed and pilot tested by additional bicultural, bilingual team members with any translation revisions made prior to the start of the study. We did not conduct psychometric testing or validation for translated survey questions.

We examined the responses to the two housing-related problems questions separately – housing instability [20] and housing condition related issues [21]. Food insecurity status was assigned based on one or more affirmative answers (no food insecurity = score of 0; food insecurity = score of 1 or 2) based on official scoring recommendations from the U.S. Department of Agriculture [22, 23] Each of four questions to screen for interpersonal violence was on a 5-point frequency scale (never = 1, rarely = 2, sometimes = 3, fairly often = 4, and frequently = 5) [24]. The sum of the numeric value of question responses was calculated for each respondent (range 4–20) and a positive screen for interpersonal violence was a score of 11-20 [24]. For the family and community support domain, we examined the two questions separately – needing support with daily activities composed of both activities of daily living (ADLs) and instrumental activities of daily living (IADLs) [25] and social isolation [26] as another.

### Analysis

We first examined the sociodemographic characteristics of the study population (age, sex and language of survey) and then prevalence of each social need. We operationalized age as a dichotomous variable (< 65 years vs. 65 + years) to compare social needs between young/middle-aged adults and older adults since eligibility for U.S. governmental and community resources can be restricted at this age cut-off. Sex was also analyzed as a binary variable (female vs. male). Language was operationalized as a multi-leveled categorical variable (English vs. Chinese vs. Spanish) based on the version of the survey completed by each respective respondent. We performed bivariate analysis assessing associations between social needs outcomes and sociodemographic characteristics (age, gender, and language of survey), as our predictors of interest, using chi-square or Fisher’s exact test. Finally, we then used multivariable logistic regression models for each HSRN, adjusting for age (< 65 years vs. 65 + years), sex and language of survey. Statistical significance was assessed at the 0.05 level. The statistical analyses were performed using STATA 17 (StataCorp, 2017; College Station, TX).

For each domain of social needs, missing datapoints were excluded in the analysis for the specific domain. However, no more than 5% of data were missing for each specific HRSN domain with 6 out of 9 domains having less than 3% missingness. In addition, we looked at associations between missingness of each domain and age, gender, and language. For 8 out of the 9 domains, there were no association with age and gender. One outcome was more likely to be missing for male respondents, and 5 outcomes were more likely to be missing for Chinese- and Spanish-speaking respondents. However, even in these cases when male or non-English speaking respondents were more likely to have a missing outcome, the missingness was significantly low for those subgroups (i.e., less than 9 participants or less than 10% of the subgroup for all the cases).

## Results

We had a total of 679 survey respondents. The mean age of respondents was 58.1 ± 17.9 years old (range 18–101) with 42.1% of respondents age 65 or older. The study population was 56.5% female, and 9.7% of respondents (n = 66) completed the survey in Chinese and 4.7% of respondents (n = 32) completed the survey in Spanish.

Table 1 presents the prevalence of social needs for the study population. The most prevalent social needs were financial strain (34.7%), having at least one housing condition related issue (26.8%), and food insecurity (22.8%). The least prevalent social need was positive screen for interpersonal violence (3.2%). About 54% of the respondents reported at least one social need.

**Table 1:** Prevalence of unmet health-related social needs (HRSNs) among respondents from an academic adult primary care practice in San Francisco, California (n = 679)

	Prevalence	Missing
Housing instability, overall, % Worried about losing steady place Do not have a steady place to live	9.7% 8.7% 1.0%	1
At least 1 housing condition related issue, % Pests Mold Lead paint/pipes Lack of heat Oven or stove not working Smoke detectors missing/not working Water leaks	27.1% 12.8% 13.3% 2.8% 5.3% 2.1% 2.8% 6.6%	8
Food insecurity, %	22.8%	7
Transportation problems, %	11.0%	9
Utility help needs, overall, % Yes Already shut off	6.1% 5.8% 0.3%	19
Positive screen for interpersonal violence (score 11–20), %	3.2%	28
Financial strain, overall, % Somewhat hard Very hard	34.7% 27.4% 7.3%	22
Needing support with daily activities, overall, % Could use a little more help Need a lot more help	13.2% 8.8% 4.4%	22
Social isolation, overall, % Often Always	8.9% 6.3% 2.6%	18
At least 1 unmet HRSN, %	53.8%	0
Number of unmet HRSNs^, % 1 unmet need 2 unmet needs 3 unmet needs 4 unmet needs 5 unmet needs 6 unmet needs 7–9 unmet needs	20.6% 11.9% 9.1% 5.0% 3.1% 2.2% 1.8%	0

In bivariate analyses presented in Table 2, prevalence of social needs significantly varied by sex, age or survey language. Female respondents were more likely to report food insecurity (25.8% versus 18.8%, p = 0.034), utility help needs (8.2% versus 3.6%, p = 0.017), positive screen for interpersonal violence (4.4% versus 1.4%, p = 0.038), financial strain (37.9% versus 30.3%, p = 0.043), needing support with daily activities (15.5% versus 9.9%, p = 0.035) and social isolation (11.1% versus 6.3%, p = 0.035) compared to male respondents. Respondents younger than 65 years old were more likely to report at least one housing condition related issue (31.5% versus 19.3%, p < 0.001) and financial strain (39.3% versus 28.2%, p = 0.003). When examining the prevalence of social needs by survey language, respondents that completed the survey in Spanish were more likely to report food insecurity (48.4% versus 21.1% (English) and 25% (Chinese), p = 0.002), transportation problems (25.8% versus 10.9% (English) and 4.9% (Chinese), p = 0.013) and needing support with daily activities (36.7% versus 12.0% (English) and 13.1% (Chinese), p = 0.002). Overall, female respondents (57.8% versus 48.8% male respondents, p = 0.021) and respondents age less than 65 (58.2% versus 46.9% age 65+, p = 0.004) were more likely to report at least one unmet HRSN.

**Table 2:** Prevalence of unmet health-related social needs (HRSN) by sex (female vs. male), age (18–64 years old vs. 65 + years old), and survey language (English vs. Chinese vs. Spanish) among 679 respondents from an academic adult primary care practice in San Francisco, California

	Female (n = 372)	Male (n = 287)	Age 18–64 (n = 378)	Age 65+ (n = 275)	English (n = 581)	Chinese (n = 66)	Spanish (n = 32)
**Housing instability, %**	11.0%	7.3%	10.3%	8.0%	9.1%	13.6%	12.5%
**At least 1 housing condition related issue, %**	29.3%	23.0%	**31.5%^**	**19.3%^**	26.5%	25.8%	34.4%
**Food insecurity, %**	25.8%*	18.8%*	24.3%	19.8%	21.1%**	25.4%**	48.4%**
**Transportation problems, %**	12.2%	9.6%	12.0%	9.2%	10.9%*	4.9%*	25.8%*
**Utility help needs, %**	8.2%*	3.6%*	6.7%	4.9%	6.2%	4.8%	6.5%
**Positive screen for interpersonal violence, %**	4.4%*	1.4%*	3.5%	2.3%	3.6%	1.7%	0%
**Financial strain, %**	37.9%*	30.3%*	39.3%**	28.2%**	34.6%	30.2%	45.2%
**Needing support with daily activities, %**	15.5%*	9.9%*	13.1%	12.9%	12.0%**	13.1%**	36.7%**
**Social isolation, %**	11.1%*	6.3%*	10.1%	6.6%	9.4%	4.7%	10.0%
**At least 1 unmet HRSN, %**	57.8%*	48.8%*	58.2%**	46.9%**	52.8%	54.6%	68.8%

* p < 0.05

** p < 0.005

^ p < 0.001

Table 3 shows the multivariable models for each social need and for the outcome at least one social need, adjusting for age, sex and language of survey. Respondents completing the survey in Spanish had significantly higher odds of reporting food insecurity (adjusted odds ratio (AOR) 3.98, 95%CI 1.87, 8.50), transportation problems (AOR 3.14, 95%CI 1.32, 7.44), and needing for support with activities of daily living (AOR 4.48, 95%CI 2.00, 10.02) compared to respondents completing the survey in English. Even after adjusting for age and language of survey, female respondents had a higher odds of reporting utility help needs (AOR 2.60, 95%CI 1.21, 5.60), positive screen for interpersonal violence (AOR 3.10, 95%CI 1.02, 9.48), financial strain (AOR 1.44, 95%CI 1.02, 2.01), and social isolation (AOR 1.90, 95%CI 1.04, 3.44) than their male counterparts. Respondents younger than age 65 had about twice the odds of at least having one housing condition related issue (AOR 2.10, 95%CI 1.41, 3.12) compared to respondents age 65 and older. Both female and younger respondents age less than 65 had significantly higher odds of reporting at least one unmet HRSN (AOR 1.46, 95%CI 1.06, 2.00; AOR 1.74, 95%CI 1.74, 2.44, respectively).

**Table 3:** Adjusted odds ratios with 95% confidence intervals of unmet health-related social needs (HRSN) by age, sex and survey language

	Age 18–64 (ref. 65+)	Female (ref. Male)	Chinese survey language (ref. English)	Spanish survey language (ref. English)
**Housing instability**	1.59 (0.87, 2.93)	1.42 (0.81, 2.48)	1.97 (0.82, 4.71)	1.21 (0.35, 4.17)
**At least 1 housing condition related issue**	**2.10 (1.41, 3.12)**	1.40 (0.97, 2.01)	1.32 (0.68, 2.55)	1.55 (0.69, 3.45)
**Food insecurity**	1.47 (0.97, 2.23)	1.45 (0.98, 2.15)	1.57 (0.80, 3.05)	**3.98 (1.87, 8.50)**
**Transportation problems**	1.26 (0.73, 2.16)	1.27 (0.76, 2.14)	0.52 (0.15, 1.80)	**3.14 (1.32, 7.44)**
**Utility help needs**	1.43 (0.69, 2.95)	**2.60 (1.21, 5.60)**	0.93 (0.26, 3.34)	1.05 (0.24, 4.66)
**Positive screen for interpersonal violence (score 11–20)**	1.44 (0.52, 4.00)	**3.10 (1.02, 9.48)**	0.59 (0.07, 4.86)	1.00 (1.00, 1.00)
**Financial strain**	1.75 (1.22, 2.52)	**1.44 (1.02, 2.01)**	1.13 (0.62, 2.08)	1.78 (0.84, 3.77)
**Needing support with daily activities**	1.09 (0.66, 1.81)	1.52 (0.93, 2.48)	1.22 (0.53, 2.82)	**4.48 (2.00, 10.02)**
**Social isolation**	1.49 (0.81, 2.73)	**1.90 (1.04, 3.44)**	0.61 (0.17, 2.11)	1.13 (0.33, 3.92)
**At least 1 unmet HRSN**	**1.74 (1.25, 2.44)**	**1.46 (1.06, 2.00)**	1.44 (0.82, 2.52)	2.24 (1.00, 5.06)

## Discussion

Our assessment of HRSNs in a convenience sample of linguistically diverse adult patients from a large urban academic primary care practice in the U.S. demonstrated a high overall prevalence of unmet social needs that can influence health. Over half of respondents had at least one unmet social need and about one in four patients reported food insecurity. In our study population, priority groups with unmet needs included patients that completed the survey in Spanish.

In our study with respondents from an adult patient population that is highly insured (~ 3% reported self-pay) with a mix of governmental (e.g. Medicare, Medicaid) and private payers, about 54% reported at least one unmet social need from the nine HRSNs included in the survey. Similarly, in one U.S. survey of unmet HRSNs of patients with private, governmental and no insurance, the overall percentage of respondents with at least one unmet social need was 48% [27]. This suggests that patients with any type of insurance payer could have unmet social needs; implementation of HRSN screening for all patients (as opposed to select subgroups) in clinical settings would best identify patients with needs and could promote equity without creating or widening disparities.

Respondents age less than 65 had a higher odds of reporting at least one unmet need than respondents age 65 + after adjusting for sex and language of survey. In unadjusted bivariate analyses, those age less than 65 had a higher prevalence of each of all nine HRSNs measured in the survey. While the high cost of living in the areas in which the study respondents live [28] may be a significant contributor to this finding, our study may underscore that HRSN screening across all ages would likely be most optimal. Referral to existing resources available for all age groups as well as referral to resources that are language concordant to patients’ language preferences is essential for patients with identified unmet social needs. Barriers to access and use of existing community and governmental resources may include language barriers to enrollment, complexity of eligibility and enrollment procedures, and qualifying factors (e.g., age, income) that affect eligibility [29–31].

The findings of this HRSN assessment within this primary care practice (which did not have a systematic social needs screening strategy) has led to interventions incorporating community engagement or a population health approach. The high prevalence of food insecurity identified in this project as well as another study focused on food insecurity among older adults with multimorbidity [4] at the same study sites contributed to the creation of a referral-based weekly food pharmacy within the clinic premises initiated in collaboration with the local city food bank and department of public health with private foundation funding. The food pharmacy offers fresh fruit and vegetables as well as protein foods, tailored to the cultural preferences of the patient population. Additionally, clinic administrative and medical leaders took interest in addressing transportation barriers (overall prevalence of 11%) as an approach to help reduce no show rates within the clinic (overall clinic no show rate ~ 13%). In response, a taxi voucher program to provide taxi vouchers to attend in person clinic visits for patients with transportation barriers and a high no show rate has been under development with administrative, medical and nursing leaders within the clinic. Our findings, paired with population-level no show data, have supported identification and outreach to populations, such as patients that speak Spanish, that may have more transportation needs.

Through an international lens, the World Health Organization has promoted building evidence for action in countries of all levels of income to address unmet social needs including food insecurity, housing and social isolation to advance health equity [32]. Globally, primary care has been identified as an essential site for delivery of social care but also have similarly lagged in integration into existing clinical workflows due to myriad of factors including insufficient structural resources to screen and intervene [33–36]. The burden of specific social needs can vary by sampled population within countries of different levels of income. For example, a cross-sectional study of food insecurity in two hospitals in Brisbane, Australia identified a household food insecurity prevalence of 41% in their study population [36] while a multistage sample and interviewer-administered household survey in the Vietnamese Mekong Delta identified a household food insecurity prevalence of 34%, [37] in contrast to our study which identified that one in four respondents reported food insecurity in San Francisco. With the COVID-19 pandemic, health, economic and social inequities including unmet social needs have even become more apparent and should be international public health priorities.

Study limitations include a sample size that limits power, limited generalizability of a random convenience sample from one primary care practice and the lack of data linking to markers of chronic disease care and health care utilization. Strengths include a linguistically diverse sample, measurement of 9 social needs and an interprofessional project team.

## Conclusion

The burden of unmet social needs within this academic adult primary care practice in an urban setting in California was substantial and compelling to support a case for social care integration in adult primary care delivery. Our findings have facilitated the development of clinic-specific stakeholder-engaged interventions to address prevalent unmet needs within the practice as well as support the broader case to advance the integration of social need screening and care into healthcare delivery including the capability to capture social need data in the electronic health record. Integration of social need screening and care in primary care delivery could advance public health efforts for health equity and social justice within a patient-centered medical home.

## Data Availability

The datasets used and analyzed during the current study are available from the corresponding author on reasonable request.

## Abbreviations

HRSN: Health-related social need
U.S.: United States
REDCap: Research Electronic Data Capture
AOR: Adjusted odds ratio
